# The Cyclic Di-GMP Receptor HpoR Modulates Mycobacterial Multidrug Susceptibility by Regulating IniBAC-Mediated Envelope Permeability

**DOI:** 10.3390/microorganisms14071579

**Published:** 2026-07-20

**Authors:** Xiao Liu, Xiaocui Ling, Kun Wang, Jiachen Zheng, Hao Li, Minhao Guo, Yanzhe Ou, Jie Lu, Weihui Li

**Affiliations:** 1 State Key Laboratory for Conservation and Utilization of Subtropical Agro-Bioresources, Guangxi Technology Innovation Center for Microbial Resources Development and Utilization, College of Life Science and Technology, Guangxi University, Nanning 530004, China; 2 College of Life Science and Technology, Central South University of Forestry and Technology, Changsha 410004, China

**Keywords:** c-di-GMP, transcriptional regulation, envelope permeability, multidrug susceptibility, mycobacteria

## Abstract

Isoniazid (INH) targets cell wall biosynthesis and is a potent antimycobacterial agent. Elucidating the regulatory networks that govern drug susceptibility in mycobacterial models is fundamental to understanding intrinsic resistance in pathogenic species. The *iniBAC* operon plays a crucial role in INH tolerance and envelope permeability, yet the transcriptional regulatory mechanisms controlling its expression in response to INH-induced stress remain incompletely understood. The second messenger cyclic di-GMP (c-di-GMP) regulates drug susceptibility in several bacteria, but its downstream receptors and regulatory pathways in mycobacteria have not been explored. Here, we demonstrate that c-di-GMP reduces INH susceptibility via the receptor HpoR. Mechanistically, c-di-GMP alleviates HpoR-mediated repression of the *iniBAC* operon in a concentration-dependent manner, which decreases envelope permeability and consequently modulates multidrug susceptibility in both *M. bovis* BCG and *M. smegmatis*. This regulatory paradigm is likely conserved in pathogenic mycobacteria.

## 1. Introduction

Tuberculosis remains one of the leading causes of death from a single infectious disease worldwide [1], and the treatment continues to receive substantial attention. First-line antituberculosis drugs, such as isoniazid (INH) and ethambutol (EMB), are effective against drug-susceptible *Mycobacterium* tuberculosis [2]. However, the emergence of multidrug-resistant *M. tuberculosis* strains has underscored the need for a deeper understanding of resistance mechanisms to identify novel drug targets [3,4,5]. The efficacy of INH and EMB suggests that targeting bacterial cell wall biosynthesis remains a promising therapeutic strategy [6,7]. However, the molecular mechanisms and regulatory networks of antibiotic resistance in mycobacteria remain poorly defined.

The *iniBAC* operon, originally identified as an INH-inducible cluster in *M. tuberculosis*, is involved in the response to external INH stress [8]. It also responds nonspecifically to various cell wall damage signals, contributing to cell wall stability [9]. The *iniB* gene shares homology with genes encoding cell wall proteins, while IniA contains a phosphopantetheine attachment site motif, suggesting it functions as an acyl carrier protein that may act as a pump-like protein to confer INH tolerance and mediate membrane fission to maintain membrane integrity [8,10,11]. The *iniC* gene encodes a protein that is 34% identical to IniA [8]. Recent studies have demonstrated that *iniBAC* operon expression is upregulated upon extensive cell wall damage and is regulated by the transcription factor IniR [12,13,14,15,16,17]. However, the transcriptional regulatory mechanisms underlying *iniBAC* function in cells remain unclear.

Cyclic di-GMP (c-di-GMP), which is synthesized from two molecules of guanosine triphosphate (GTP), is a highly conserved second messenger in bacteria [18,19,20]. It regulates diverse bacterial physiological processes, including drug resistance [21]. Inhibition of the diguanylate cyclase DgcM and modulation of c-di-GMP levels can inhibit antibiotic resistance to beta-lactams in *Escherichia coli* [22]. The virulence factor pyocyanin and c-di-GMP jointly regulate biofilm formation and multidrug efflux pump expression through the common receptor BrlR in *Pseudomonas aeruginosa* [23,24]. Transcriptional regulation is central to bacterial adaptation to environmental and external pressures [25], making it a valuable entry point for understanding drug susceptibility mechanisms in mycobacteria. However, whether c-di-GMP is involved in the response to INH stress in mycobacteria has not been reported.

HpoR is an essential transcriptional regulator in mycobacteria, with a wide range of downstream target genes. We previously reported that the expression of a redox cluster is induced by HpoR in response to elevated levels of c-di-GMP [26]. As a transcription repressor, HpoR binds to the 12 bp palindromic sequence motif in the upstream regulatory region of the redox cluster operon, exerting an inhibitory effect. HpoR binds c-di-GMP in a 1:1 manner and is considered a mediator of c-di-GMP-regulated H_2_O_2_ resistance in mycobacteria [26]. Another c-di-GMP receptor, LtmA, has a similar DNA-binding motif and can physically interact with HpoR to participate in antioxidant defense in mycobacteria. Notably, while HpoR represses target gene transcription, LtmA promotes expression of redox clusters [27]. Whether HpoR also contributes to INH susceptibility remains unclear.

Here, we report that c-di-GMP modulates *iniBAC* expression by influencing the DNA-binding activity of HpoR to the *iniBAC* promoter, thereby regulating envelope barrier function and likely contributing to protection of the vaccine strain *M. bovis* BCG against multidrug stress. Given the high sequence identity of HpoR and the *iniBAC* promoter between *M. bovis* BCG and *M. tuberculosis*, this study establishes a conserved regulatory model in mycobacteria, which may offer mechanistic insights into the drug tolerance of pathogenic strains.

## 2. Materials and Methods

### 2.1. Expression and Purification of Recombinant Proteins

The *hpoR* gene was amplified from the *M. tuberculosis* genome using primer pairs (Tsingke Biotech, Beijing, China) (Supplementary Table S1). Polymerase chain reaction (PCR) was performed in a 50 μL reaction (Ex Taq^®^ DNA Polymerase Mix (#RR001B, TaKaRa, Shiga, Japan)) with predenaturation at 95 °C for 5 min, 30 amplification cycles (95 °C for 10 s, optimal primer-specific temperature for 30 s, and 72 °C for 30 s), and a final extension step at 72 °C for 5 min. The amplified DNA fragment was cloned into a modified pET-SUMO expression vector or pMV261 overexpression vector to generate recombinant plasmids. *E. coli* BL21 cells transformed with the recombinant plasmid were grown to an optical density at 600 nm (OD_600_) of 0.6 in 200 mL of LB medium (Tryptone 20 g/L (#LP0042B, OXOID, Basingstoke, UK), Yeast Extract 10 g/L (#LP0021B, OXOID, UK), NaCl 20 g/L (#A501218, Sangon Biotech, Shanghai, China)). Protein expression was induced by the addition of 0.3 mM isopropyl β-D-1-thiogalactopyranoside (IPTG) (#I8070, Solarbio, Beijing, China) at 16 °C for 12 h. The obtained bacteria were resuspended and sonicated in buffer (100 mM Tris-HCl) (#T1503, Sigma, Merck KGaA, Darmstadt, Germany) (pH 7.5), 500 mM NaCl, and 10 mM imidazole (#I8090, Solarbio, China), and the lysates were centrifuged at 9000× *g* for 30 min. The clarified supernatant was loaded onto the Ni-NTA agarose (#SA05101L, Smart-Lifesciences, Changzhou, China) affinity column and washed with wash buffer (100 mM Tris-HCl (pH 8.0), 500 mM NaCl, and 100 mM imidazole). Proteins were then eluted using elution buffer (100 mM Tris-HCl (pH 8.0), 500 mM NaCl, and 250 mM imidazole). The eluate was then dialyzed overnight in buffer (20 mM Tris-HCl, 100 mM NaCl, 1 mM DL-dithiothreitol (DTT (#D8220, Solarbio, China)), 10% glycerol (#A600232, Sangon Biotech, China) [28]. After dialysis, the protein was stored at −80 °C. Purified proteins were verified by sodium dodecyl sulfate-polyacrylamide gel electrophoresis (SDS-PAGE). Protein concentration was detected by the NanoDrop (Thermo Fisher, Waltham, MA, USA).

### 2.2. Proteomic Analysis

*Mycobacterium bovis* BCG strains were cultured in 7H9 medium supplemented with 0.5% (*v*/*v*) glycerol and 0.05% (*v*/*v*) Tween-80 at 37 °C with shaking at 160 rpm to mid-log phase (OD~600~ = 1.2). Cells were harvested by centrifugation (3350× *g*, 5 min), washed twice with PBS, and flash-frozen in liquid nitrogen. Protein extraction was performed by sonicating the samples three times on ice in lysis buffer (8 M urea, 1% protease inhibitor cocktail) using a high-intensity ultrasonic processor, followed by centrifugation at 12,000× *g* for 10 min at 4 °C to remove debris. Protein concentrations were determined using a BCA kit according to the manufacturer’s instructions. For digestion, protein samples were reduced with 5 mM dithiothreitol at 56 °C for 30 min, followed by alkylation with 11 mM iodoacetamide in the dark at room temperature for 15 min. The samples were then diluted with 100 mM TEAB to a final urea concentration of less than 2 M. Trypsin was added at a 1:50 enzyme-to-protein ratio for the first overnight digestion, followed by a second digestion for 4 h at a 1:100 ratio. The resulting peptides were desalted using a C18 SPE column. Desalted peptides were dissolved in mobile phase A (0.1% formic acid and 2% acetonitrile) and separated using a Nano Elute UHPLC system (Thermo Fisher, Waltham, MA, USA) with mobile phase B (0.1% formic acid and 100% acetonitrile) at a flow rate of 450 nL/min. The gradient was set as follows: 0–40 min, 6–24% B; 40–52 min, 24–35% B; 52–56 min, 35–80% B; 56–60 min, 80% B. The separated peptides were analyzed on a timsTOF Pro 2 mass spectrometer equipped with a Capillary ion source. The ion source voltage was set to 1.65 kV, and MS/MS spectra were acquired in parallel accumulation serial fragmentation (PASEF) mode over a mass range of *m*/*z* 100–1700. Dynamic exclusion was set to 30 s. The raw MS data were searched against the *Mycobacterium bovis* BCG protein database (BLAST_Mycobacterium_bovis_strain_BCG_410289_PR_20220526.fasta, 3891 entries) using MaxQuant (v1.6.15.0). The search parameters included: trypsin/P digestion with up to two missed cleavages; carbamidomethylation of cysteine as a fixed modification; oxidation of methionine and N-terminal acetylation as variable modifications; peptide length ≥ 7 amino acids; and precursor mass tolerance of 20 ppm for both first and main searches, with fragment mass tolerance set to 20 ppm. Both protein and peptide-spectrum match (PSM) identifications were filtered at a 1% false discovery rate (FDR). For label-free quantification, the LFQ (label-free quantification) intensities generated by MaxQuant were exported, and median normalization was applied in R (v4.2.2) to correct for sample loading variations across different runs. Differentially expressed proteins were defined by a fold change > 1.2 and a Benjamini–Hochberg-adjusted *p*-value (Q-value) < 0.05. To control the false-positive rate arising from multiple hypothesis testing, we further applied the Benjamini–Hochberg (BH) procedure to adjust the raw *p*-values, and proteins with an adjusted Q-value < 0.05 were considered statistically significant. Given that three independent biological replicates per group are standard and widely accepted for discovery-based shotgun proteomics, we further mitigated the risk of false positives by combining a stringent fold-change cutoff (1.2-fold) with the BH-adjusted Q-value (<0.05). This dual-filtering strategy effectively controls the family-wise error rate without over-penalizing true biological signals, and all significant hits were subsequently validated via orthogonal targeted assays. Functional enrichment (GO and KEGG) and protein–protein interaction analyses were carried out using the R package “clusterProfiler” (v4.6.2) and the STRING database (v12.0), respectively. Principal component analysis (PCA) and heatmap clustering were performed using the R packages “factoextra” (v1.0.7) and “pheatmap” (v1.0.12). All mass spectrometry proteomics data have been deposited to the ProteomeXchange Consortium via the PRIDE partner repository with the dataset identifier PXD039181.

### 2.3. Electrophoretic Mobility Shift Assay

The upstream regulatory sequence of the *iniBAC* operon (*iniBAC*p) and *bcg3074c*p used for EMSA were amplified by PCR from the genome of the *M. tuberculosis* strain. The reactions (20 μL) for measuring the mobility shift contained DNA fragments and various amounts of protein diluted in a buffer containing 50 mM Tris-HCl (pH 7.5), 10 mM MgCl_2_, 1 mM DTT, and 50 mM NaCl. The protein and c-di-GMP were incubated for 15 min at room temperature, and then the mixture and deoxyribonucleic acid (DNA) fragments were added to the reaction mixture and incubated for 15 min. The mixture was loaded on an 8% native polyacrylamide gel (#A1020, Solarbio, China) containing 1× glycine buffer. Electrophoresis was performed at 150 V. Images were recorded using a Fluorescence Image Analyzer (Bio-Rad, Hercules, CA, USA). The primers used in promoter amplification were shown in the Supplementary Table S1.

### 2.4. β-Galactosidase Activity Assay

The β-galactosidase activity experiment was carried out in the *M. bovis* Bacillus Calmette–Guérin (BCG) strain by constructing an operon-*lacZ* expression vector based on pMV261 [29]. The *iniBAC* promoter and control promoter were cloned into the pMV261 vector, and the reporter gene *lacZ* was cloned behind the promoter. The plasmids were electroporated into *hpoR* knockout and WT *M. bovis* BCG strains to obtain the recombinant reporter strains. Recombinant strains were grown in 7H9 medium at 37 °C until the log phase was reached. β-Galactosidase levels were measured as previously described [30]. The primers used in promoter amplification were shown in the Supplementary Table S1. Each sample was analyzed in three biological replicates and two-tailed Student’s *t*-tests were performed for statistical analysis.

### 2.5. Chromatin Immunoprecipitation Assay

*M. bovis* BCG strains were grown to mid-log phase in 7H9 medium and incubated with 1% formaldehyde (#A501912, Sangon Biotech, China), and the reaction was stopped with 0.125 M glycine (#62011516, Sinopharm, Beijing, China). Cross-linked cells were then collected and resuspended in 1 mL of Tris-buffered saline with Tween 20, 20 mM Tris-HCl, 150 mM NaCl, 0.1% Tween 20 (#A600560, BBI, Shanghai, China), 0.1% Triton X-100 (#A110694, Dimond), pH 7.5). The obtained samples were sonicated for 10 min (200 W, sonicate for 2 s, then pause for 2 s) at 4 °C. Treated samples were centrifuged to collect supernatant extracts. 500 µL of supernatant was saved as the input sample. Commercial His-tag antibody (#CSB-MA000011M0m, CUSABIO, Wuhan, China) or preimmune mouse serum (#NS03L, Sigma, Darmstadt, Germany) was added to the sample extract and incubated for 3 h at 4 °C with shaking. The complexes were immunoprecipitated with 50 μL of 50% protein A-agarose (#17127901, GE) for 1 h at 4 °C. Immune complexes were recovered by centrifugation and resuspended in 100 μL of TE (20 mM Tris-HCl (pH 7.8), 10 mM ethylenediaminetetraacetic acid, 0.5% sodium dodecyl sulfate). The samples were then incubated with 10 μg/mL proteinase K for 6 h at 65 °C. The DNA was recovered from the immunocomplex as the sample of ChIP and P [31]. The samples of Input, I and P were analyzed by conventional PCR (25 μL reaction for 35 cycles) and real-time qPCR using 20 μL SYBR qPCR Green Master Mix (#PC3301, Aidlab, Beijing, China) on QuantStudio 3 Real-Time PCR System (Thermo Fisher, USA) with *iniBAC*p primers (Supplementary Table S1). Each sample was analyzed in three technical replicates; the data were calculated using the 2^−ΔΔCt^ method, and two-tailed Student’s *t*-tests were performed for statistical analysis.

### 2.6. RT-qPCR Assay

A real-time quantitative polymerase chain reaction (RT-qPCR) assay was performed to characterize the expression of target genes. Mycobacterial cells grown in 7H9 medium (OD_600_ 1.0–1.2) were harvested for RNA extraction using an RNA extraction kit (#RN0802, Aidlab, China). Total ribonucleic acid (RNA) (500 ng) was reverse transcribed to cDNA following the RNA reverse transcription protocol, and cDNA was used as a template for RT-qPCR analysis using a SYBR qPCR Green Master Mix. The degrees of change in the levels of genes were calculated using the 2^−ΔΔCt^ method; each sample was analyzed in three technical replicates and two-tailed Student’s *t*-tests were performed for statistical analysis. The primers used for RT-qPCR analysis are shown in Supplementary Table S1.

### 2.7. Determination of Mycobacterial Growth

To determine the differences in mycobacterial growth under INH stress, the recombinant strains were transferred to 7H9 medium and cultured to the log phase in a shaker at 37 °C. Mycobacterial cells were collected, and the bacterial cell density was adjusted to an OD_600_ of 0.5 in fresh 7H9 medium, followed by a 10-fold serial dilution 3 times. Then, 2 μL of each dilution was spotted on 7H10 solid media supplemented with 0, 0.07 or 0.1 μg/mL INH, and the plates were cultivated at 37 °C for 15 days. The growth of each recombinant strain under INH stress was observed. To determine the effects of INH, EMB, RIF and Str, mycobacteria were inoculated in 7H9 media supplemented with the respective drugs and incubated at 37 °C and 160 rpm. One milliliter of sample was collected and diluted in 7H9 medium, followed by plating the dilutions on 7H10 plates at different time points. Single colonies were counted on the plates in three technical replicates. Two-tailed Student’s *t*-tests were performed for statistical analysis.

### 2.8. Construction of ydeH- and iniBAC-Overexpressing Mycobacteria

The *ydeH* and *ydeH*(mut) genes were amplified from the *Escherichia coli* genome using the corresponding primer pairs. The *iniBAC* operon was amplified from the *M. bovis* BCG genome. The amplified *ydeH* and *ydeH*(mut) fragments were cloned into the pMV261 plasmid, while the amplified *iniBAC* fragment was cloned into the pJAM2 plasmid. Subsequently, these recombinant plasmids were transferred into *M. bovis* BCG or *M. smegmatis* by transformation. The primers used for gene overexpression are shown in Supplementary Table S1.

### 2.9. Measurement of c-di-GMP in the M. bovis BCG Strain

The *ydeH*(mut)- and *ydeH*-overexpressing *M. bovis* BCG strains were cultured to the mid-log phase for the determination of the c-di-GMP levels using a modified protocol [32]. The cells were collected and washed twice with phosphate-buffered saline (PBS). Subsequently, the cells were suspended in 10 mL of ddH_2_O and ultrasonicated at 450 W for 1 h. The supernatant was collected after centrifugation and was extracted with phenol/chloroform and concentrated to 1 mL. Finally, the c-di-GMP concentration was quantified using an enzyme-linked immunosorbent assay (ELISA) kit (#F20205-B, FANKEWEI, Shanghai, China). It should be noted that ELISA-based c-di-GMP quantification relies on antibody-based recognition and is subject to potential cross-reactivity with structurally related nucleotides (e.g., cGMP, cAMP, and GTP) present in crude bacterial lysates, as well as matrix interference from cellular components. Consequently, the c-di-GMP levels obtained by this method are considered relative quantifications rather than absolute concentrations. For precise absolute quantification, liquid chromatography-tandem mass spectrometry (LC-MS/MS) with stable isotope-labeled internal standards remains the reference standard, although it was not employed in the current study. To investigate the effect of INH stress on the intracellular c-di-GMP level in *M. bovis* BCG, mycobacterial cells of the WT and *ydeH*-overexpressing strains were collected and treated with 10 μg/mL INH or without INH for 8 h at 37 °C. Each strain was analyzed in three biological replicates and two-tailed Student’s *t*-tests were performed for statistical analysis.

### 2.10. Measurement of Envelope Permeability by the DPH-Binding Assay

The envelope permeability of *M. bovis* BCG and *M. smegmatis* was assessed using permeation by 1,6-diphenyl-1,3,5-hexatriene (DPH) (#1720-32-7, Solarbio, China). The mycobacteria were cultured to the logarithmic phase, and the cells were collected and washed twice with PBS. Then, the bacterial cells were resuspended in PBS buffer containing 2.5 μM DPH and incubated at 37 °C for 30 min in the dark. The absorbance at 600 nm and the fluorescence intensity were measured using a TECAN Infinite M200 Pro Nano Quant microplate reader (Mannedorf, Männedorf, Switzerland) at an emission wavelength of 430 nm and an excitation wavelength of 358 nm. The relative fluorescence intensity was calculated using RFU/OD_600_. Error bars represent averages of three independent biological replicates, and a two-tailed Student’s *t*-test was performed for statistical analysis.

## 3. Results

### 3.1. INH Susceptibility of an M. bovis BCG Strain with a High Level of c-di-GMP

We previously showed that c-di-GMP integrates the two opposite functions of the transcription factors LtmA and HpoR to aid oxidative defense in mycobacteria [27]. To explore whether c-di-GMP affects other stress-resistance pathways, we electroporated the wild-type *M. bovis* BCG strain with plasmids overexpressing the *E. coli* diguanylate cyclase gene ydeH or its catalytically inactive mutant *ydeH*(mut), generating the high-c-di-GMP strain (BCG/*ydeH*) and the control strain (BCG/*ydeH*(mut)), respectively. ELISA-based intracellular c-di-GMP detection revealed that overexpression of *ydeH* led to relatively higher levels of c-di-GMP in the *M. bovis* BCG strain compared to the mutant control (Figure S1), consistent with the established DGC activity of YdeH. Given the semi-quantitative nature of the ELISA, these results primarily reflect relative differences in c-di-GMP abundance between strains.

**Figure 1:**
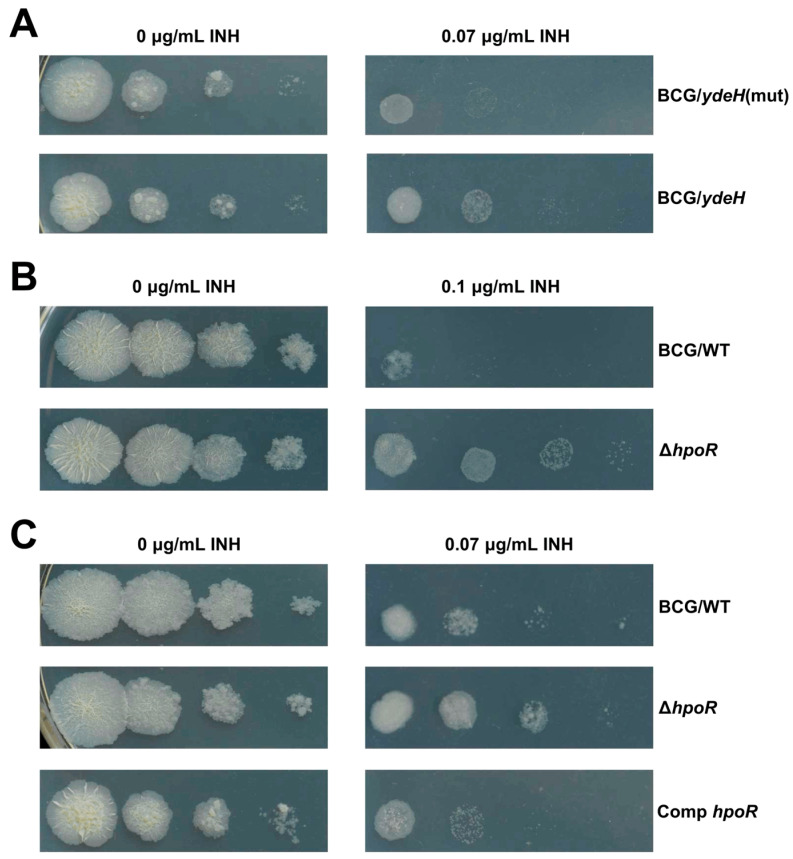
Effects of elevated levels of c-di-GMP and HpoR on the INH susceptibility of *M. bovis* BCG. (**A**) Susceptibility of the BCG/*ydeH* and BCG/*ydeH*(mut) strains to INH. (**B**) Susceptibility of the wild-type (BCG/WT) and Δ*hpoR M. bovis* BCG strains to isoniazid (INH). (**C**) Susceptibility of the BCG/WT, Δ*hpoR*, and Comp *hpoR M. bovis* BCG strains to INH. Cells were 10-fold serially diluted and spotted on 7H10 agar plates (**left**, control) and 7H10 agar plates containing 0.07 or 0.1 μg/mL INH (**right**).

A colony-forming unit (CFU) assay of cell suspensions was conducted to investigate the effect of c-di-GMP in response to INH stress in *M. bovis* BCG. No growth difference was observed between BCG/ydeH and BCG/ydeH(mut) strains on 7H10 plates without INH. In contrast, on 7H10 plates supplemented with 0.07 μg/mL INH, growth of the BCG/ydeH strain was significantly greater than that of the BCG/ydeH(mut) strain (Figure 1A).

These findings indicate that the *M. bovis* BCG strain with relatively elevated levels of c-di-GMP exhibited reduced INH susceptibility compared to the control strain under INH stress.

### 3.2. INH Susceptibility of an hpoR Deletion Mutant

c-di-GMP can directly bind to downstream transcription factor receptors to regulate bacterial physiological processes in response to environmental stress [33]. We previously confirmed that the c-di-GMP receptor HpoR is a global transcriptional repressor [26], suggesting that the reduced INH susceptibility observed in strains with elevated c-di-GMP may be related to HpoR. CFU assay comparing the *hpoR* deletion mutant (Δ*hpoR*) and the wild-type (BCG/WT) strain showed no growth difference between the two strains. However, as INH concentration increased to 0.1 μg/mL, growth of the Δ*hpoR* strain was significantly greater than that of the BCG/WT strain (Figure 1B).

To further verify that the reduced INH susceptibility was specifically due to the deletion of *hpoR* rather than secondary mutations, an *hpoR* complementary strain (Comp *hpoR*) was constructed. CFU assay of the BCG/WT, Δ*hpoR* and Comp *hpoR* strains confirmed that the Δ*hpoR* strain exhibits reduced INH susceptibility (Figure 1C).

Taken together, these findings indicate that HpoR positively influences susceptibility to INH in *M. bovis* BCG and suggest that c-di-GMP likely regulates bacterial INH susceptibility through its receptor HpoR.

### 3.3. HpoR Negatively Regulates the Expression of iniBAC

**Figure 2:**
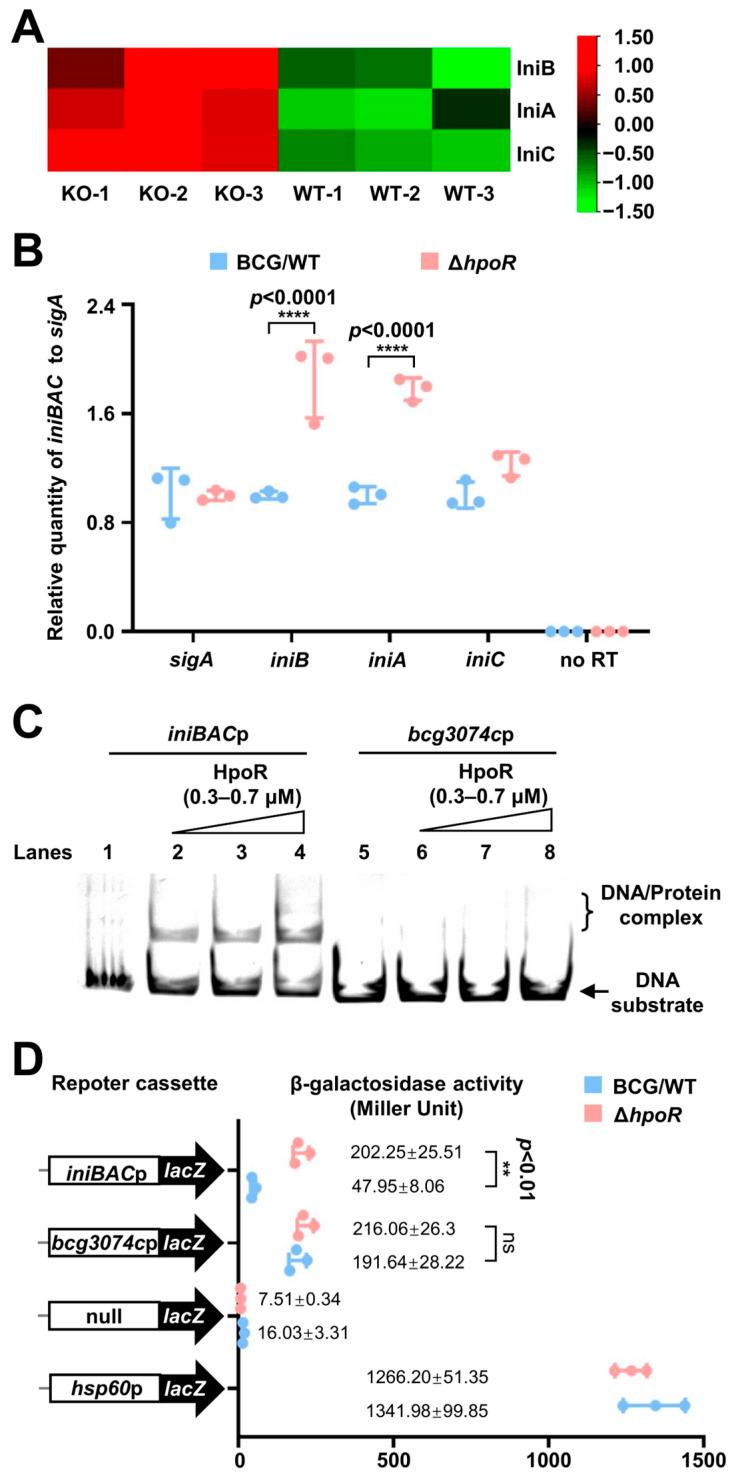
Regulatory effect of HpoR on *iniBAC* genes in *M. bovis* BCG. (**A**) Heatmap of IniBAC expression in the proteome. KO represents the differentially expressed IniBAC proteins in the *hpoR* knockout strain. WT represents the proteins expressed by the wild-type strain. Three biological replicates were performed for each sample; differential proteins were selected with a fold change > 1.2 and a Benjamini–Hochberg-adjusted Q-value < 0.05 based on three biological replicates. (**B**) Expression levels of *iniBAC* in wild-type (BCG/WT) and Δ*hpoR M. bovis* BCG strains, as determined by an RT-qPCR assay. *iniB*, *iniA*, and *iniC* were quantified using *sigA* as the standard control, and no RT was used as a genomic DNA contamination control. RT-qPCR was performed using three biological replicates, and the results were calculated using the 2^−ΔΔCt^ method. A two-tailed Student’s *t*-test was performed for statistical analysis (**** *p* < 0.0001). (**C**) Binding activities of HpoR to *iniBAC*p and *bcg3074c*p, as determined by an EMSA. DNA substrates were coincubated with 0.3–0.7 μM HpoR and loaded on the gel for analysis. Lanes 1–8: The promoter *bcg3074c*p was used as a control. (**D**) The β-galactosidase activity assay. The effect of HpoR on gene expression was assayed by constructing an *iniBAC*p-*lacZ* plasmid. The activity of β-galactosidase was further examined in the wild-type and Δ*hpoR M. bovis* BCG strains. The data are presented as Miller units (right panel). Left column: schematic representation of each clone used to generate recombinant strains. Null promoter-*lacZ*, *hsp60*p-*lacZ* and *bcg3074c*p-*lacZ* were used as controls. The values presented are the averages of three independent experiments. A two-tailed Student’s *t*-test was performed for statistical analysis (** *p* = 0.0018, ns: no significant difference).

To identify HpoR target genes associated with INH susceptibility and elucidate the underlying regulatory mechanism, we performed proteomic analysis of the *hpoR* deletion mutant. The results revealed numerous upregulated and downregulated proteins in the Δ*hpoR* strain compared to the wild-type *M. bovis* BCG strain (Figure S2, Table S2). Notably, IniB, IniA and IniC were significantly upregulated in the Δ*hpoR* strain (Figure 2A). Given that the *iniBAC* cluster is associated with INH susceptibility in *M. bovis* BCG and *M. tuberculosis* [10,17], this suggested that *iniBAC* might be a downstream target of HpoR in the regulation of INH susceptibility. To verify this hypothesis, an RT-qPCR assay was performed to test the effect of HpoR on the expression of *iniBAC*. The results showed that the *iniB* and *iniA* genes were both upregulated in the Δ*hpoR* strain (Figure 2B). Electrophoretic mobility shift assays demonstrated that HpoR (0.3–0.7 μM) could bind effectively to the *iniBAC* promoter (lanes 2–4) but not to the *bcg3074c* promoter (lanes 6–8) (Figure 2C). We further characterized the HpoR-binding motif in the *iniBAC* promoter by analyzing the conserved bases of the previously identified palindrome sequence motif of HpoR [26]. EMSA showed that a short DNA fragment (*iniBAC*p1) containing two inverted repeated palindromic motifs (CAGACNNNNNNNTGTCTG) was strongly bound by HpoR (Figure S3, lanes 2–4). However, HpoR lost the binding ability to the mutated *iniBAC*p2 (Figure S3, lanes 6–8).

Next, we conducted a β-galactosidase activity assay to characterize the regulatory effect of HpoR on the expression of *iniBAC*. A series of plasmids containing *lacZ* alone or *lacZ* regulated by *hsp60*p/*iniBAC*p*/bcg3074c*p promoters was constructed and transformed into the wild-type and *hpoR* deletion *M. bovis* BCG strains. Compared with the low expression of null promoter-*lacZ* strains, *hsp60*p markedly increased the expression of *lacZ* in the BCG/WT and Δ*hpoR* strains, confirming normal reporter system function. The expression of *iniBAC*p-*lacZ* was significantly greater in the Δ*hpoR* strain than in the BCG/WT strain, while no difference was observed for the expression of *bcg3074c*p-*lacZ* between the two strains (Figure 2D).

In summary, these results showed that the repressor HpoR affected INH susceptibility by directly binding to *iniBAC*p to negatively regulate the expression of *iniBAC* in *M. bovis* BCG.

### 3.4. IniBAC Contributes to Reduced Envelope Permeability and Multidrug Susceptibility in M. bovis BCG and M. smegmatis

**Figure 3:**
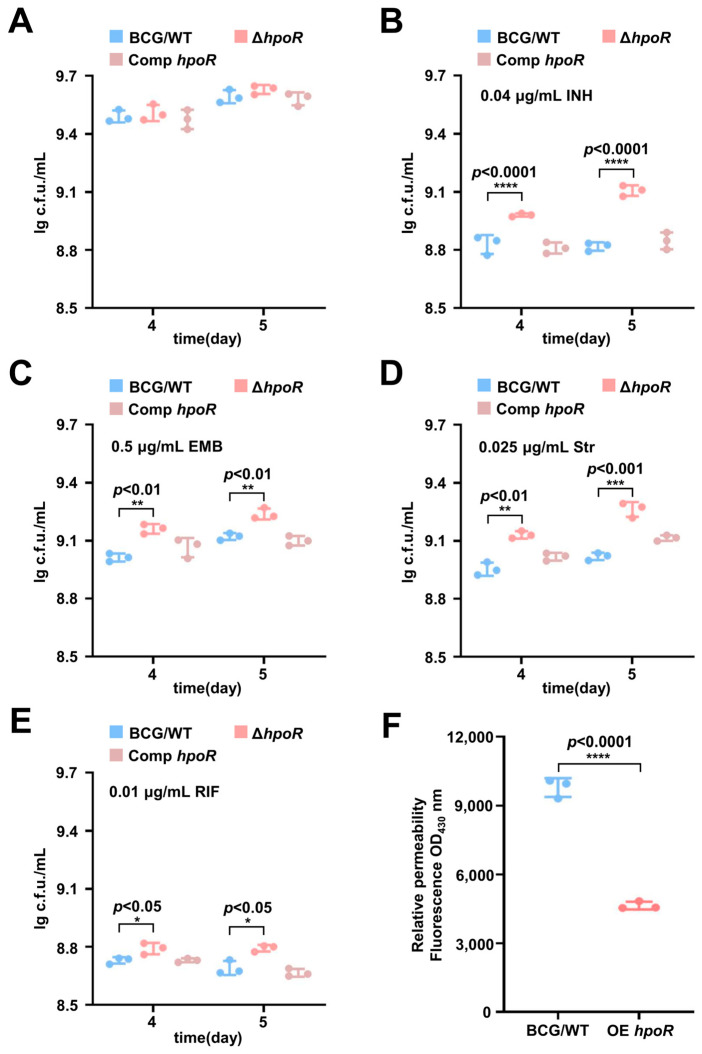
Effects of HpoR on multidrug susceptibility of *M. bovis* BCG. To determine the effects of drugs on the growth of the wild-type (BCG/WT), Δ*hpoR*, and Comp *hpoR M. bovis* BCG strains, the strains were grown in 7H9 medium (**A**) and 7H9 medium supplemented with 0.04 μg/mL INH (**B**) (**** *p* < 0.0001), 0.5 μg/mL EMB (**C**) (** *p* = 0.0013 (left), ** *p* = 0.0038 (right)), 0.025 μg/mL Str (**D**) (** *p* = 0.0014, *** *p* = 0.0006), and 0.01 μg/mL RIF (**E**) (* *p* = 0.0359 (left), * *p* = 0.0118 (right)). Mycobacterial colony-forming units (cfu) were counted on 7H10 plates. Error bars represent averages of three independent experiments, and a two-tailed Student’s *t*-test was performed for statistical analysis. (**F**) Envelope permeability of the wild-type (BCG/WT) and *hpoR*-overexpressing (OE *hpoR*) *M. bovis* BCG strains. Error bars represent averages of three independent experiments, and a two-tailed Student’s *t*-test was performed for statistical analysis (**** *p* < 0.0001).

The IniBAC proteins respond to cell wall damage signals in mycobacteria [15]. We hypothesized that the upregulation of *iniBAC* might negatively influence INH susceptibility by maintaining envelope permeability in the *hpoR*-deleted *M. bovis* BCG strain. To test our hypothesis, we first examined the growth of the BCG/WT, Δ*hpoR*, and Comp *hpoR* strains upon treatment with several drugs, including 0.04 μg/mL INH, 0.5 μg/mL EMB, 0.025 μg/mL Str and 0.01 μg/mL RIF. As shown in Figure 3A, no growth differences were observed among the three strains in a drug-free 7H9 medium. In contrast, bacterial counts of the Δ*hpoR* strain were significantly greater than those of the BCG/WT strain at both time points (4 and 5 days), while the Comp *hpoR* strain could partially or completely restore the drug susceptibility of the BCG/WT strain (Figure 3B–E). Furthermore, we tested the envelope permeability of BCG/WT and *hpoR*-overexpressing (OE *hpoR*) strains to explore the effect of HpoR on envelope permeability. Overexpression of *hpoR* significantly reduced envelope permeability (Figure 3F). 

**Figure 4:**
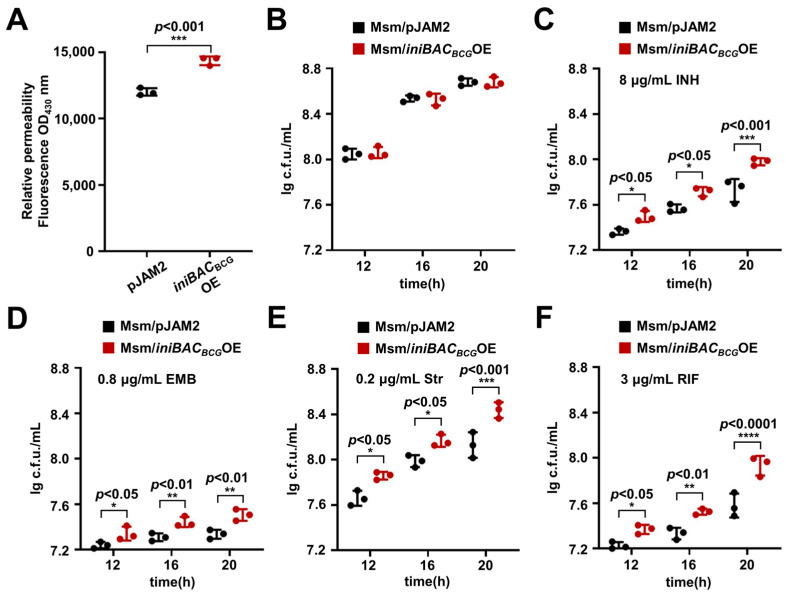
Effects of *iniBAC* on envelope permeability and drug susceptibility of *M. bovis* BCG and *M. smegmatis*. (**A**) Cell wall permeability of the *iniBAC_BCG_*-overexpressing (Msm/*iniBAC_BCG_*OE) *M. smegmatis* strain and the *M. smegmatis* strain carrying an empty pJAM2 vector (Msm/pJAM2). Error bars represent averages of three independent biological experiments, and a two-tailed Student’s *t*-test was performed for statistical analysis (*** *p* = 0.0005). (**B**–**F**) To evaluate the effects of drugs on the growth of the Msm/*iniBAC_BCG_*OE and Msm/pJAM2*,* the two strains were grown in 7H9 medium (**B**) and 7H9 medium supplemented with 8 μg/mL INH (**C**) (* *p* = 0.0298 (left), * *p* = 0.0161 (right), and *** *p* = 0.0002), 0.8 μg/mL EMB (**D**) (* *p* = 0.0495, ** *p* = 0.0097 (left), and ** *p* = 0.0016 (right)), 0.2 μg/mL Str (**E**) (* *p* = 0.0128 (left), * *p* = 0.0237 (right), and *** *p* = 0.0004) and 3 μg/mL RIF (**F**) (* *p* = 0.0496, ** *p* = 0.0092, and **** *p* < 0.0001). Mycobacterial colony-forming units (cfu) were counted on 7H10 plates. Error bars represent averages of three biological replicates, and a two-tailed Student’s *t*-test was performed for statistical analysis.

To further demonstrate that the IniBAC proteins reduce envelope permeability and drug susceptibility, we constructed a recombinant strain of *Mycobacterium smegmatis* by overexpressing *M. bovis* BCG *iniBAC* via the pJAM2 vector (Msm/*iniBAC_BCG_*OE) and a control strain (Msm/pJAM2). As shown in Figure 4A, the envelope permeability (measured by DPH fluorescence) of Msm/iniBAC*_BCG_*OE was significantly higher than that of Msm/pJAM2. We also tested growth of the two recombinant strains under 8 μg/mL INH, 0.8 μg/mL EMB, 0.2 μg/mL Str, and 3 μg/mL RIF stress. Compared to the Msm/pJAM2 strain, the Msm/*iniBAC_BCG_*OE strain showed a better growth under drug stress for 12, 16, and 20 h (Figure 4C–F), while growth was similar under no drug stress conditions (Figure 4B).

In conclusion, our data demonstrated that HpoR likely affects envelope permeability and multidrug susceptibility, potentially via its regulation of *iniBAC* by inhibiting the expression of *iniBAC* in *M. bovis* BCG and *M. smegmatis*.

### 3.5. c-di-GMP Alleviates the Inhibitory Effect of HpoR on iniBAC

**Figure 5:**
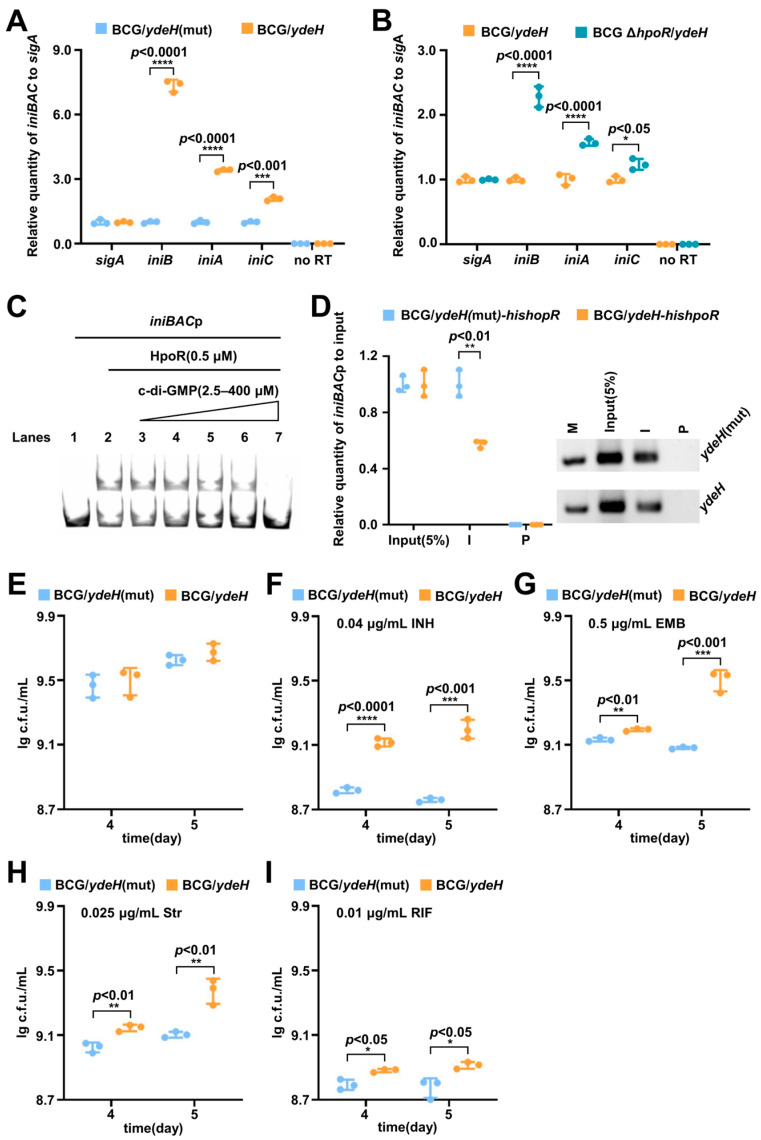
Effects of c-di-GMP on HpoR-induced regulation of the expression of the *iniBAC* genes and multidrug susceptibility in *M. bovis* BCG. (**A**) Expression levels of the *iniBAC* cluster in the BCG/*ydeH* and BCG/*ydeH*(mut) strains, as determined by an RT-qPCR assay. Quantitative analyses of *iniB*, *iniA*, and *iniC* expression were carried out using *sigA* as the control, and no RT was used as a genomic DNA contamination control. RT-qPCR was performed for three biological replicates, and the results were calculated using the 2^−ΔΔCt^ method (**** *p* < 0.0001, *** *p* = 0.0003). (**B**) Expression levels of the *iniBAC* cluster in the BCG/*ydeH* and BCG Δ*hpoR*/*ydeH* strains, as determined by an RT-qPCR assay. Quantitative analyses of *iniB*, *iniA*, and *iniC* expression were carried out using *sigA* as the control, and no RT was used as a genomic DNA contamination control. RT-qPCR was performed for three biological replicates, and the results were calculated using the 2^−ΔΔCt^ method (**** *p* < 0.0001, * *p* = 0.0379). (**C**) EMSA assay for the effect of c-di-GMP on the DNA-binding activity of HpoR. The increasing amounts of c-di-GMP (2.5–400 μM) (Lanes 2–7, Lane 1 only iniBAC promoter DNA) were added to the reactions and incubated with 0.5 μM HpoR for 15 min. Then the *iniBAC*p was added to the mixture and incubated for 15 min. (**D**) Effect of c-di-GMP on the intracellular DNA-binding activity of HpoR in *M. bovis* BCG, as determined by a ChIP assay. The input (5%) indicates that the supernatant of disrupted cells was diluted to 5% ChIP using preimmune (P) or immune (I) sera raised against HisHpoR, M, DNA Marker. These samples were used as the templates for qPCR (**left panel**) and PCR (**right panel**). Error bars represent averages of three independent experiments. A two-tailed Student’s *t*-test was performed for statistical analysis (** *p* = 0.0016). (**E**–**I**) Assay for the effect of the drug on the growth of the BCG/*ydeH* and BCG/*ydeH*(mut) *M. bovis* BCG strains. Two strains were grown in 7H9 medium (**E**) and 7H9 medium supplemented with 0.04 μg/mL INH (**F**) (**** *p* < 0.0001, *** *p* = 0.0002), 0.5 μg/mL EMB (**G**) (** *p* = 0.0020, *** *p* = 0.0004), 0.025 μg/mL Str (**H**) (** *p* = 0.0047 (left), ** *p* = 0.0043 (right)), 0.01 μg/mL RIF (**I**) (* *p* = 0.0103 (left), * *p* = 0.0189 (right)). Mycobacteria colony-forming units (cfu) were counted on 7H10 plates. Error bars represent averages of three independent experiments. A two-tailed Student’s *t*-test was performed for statistical analysis.

HpoR is a receptor of c-di-GMP in mycobacteria [26], and *iniBAC* is the target gene cluster of HpoR in the regulation of drug susceptibility. We therefore sought to confirm whether c-di-GMP is also involved in the transcriptional regulatory pathway through which HpoR regulates the expression of *iniBAC*. RT-qPCR showed that the expression of the *iniBAC* cluster was markedly upregulated in the BCG/*ydeH* (containing high levels of c-di-GMP) strain compared with the BCG/*ydeH*(mut) strain (Figure 5A). Furthermore, to demonstrate that c-di-GMP regulates the expression of *iniBAC* through the transcriptional regulator HpoR, we constructed an *ydeH*-overexpressing strain based on the *hpoR* deletion strain (BCG Δ*hpoR*/*ydeH*). RT-qPCR results showed that the expression levels of *iniBAC* in the BCG Δ*hpoR*/*ydeH* strain were greater than those in the BCG/*ydeH* strain (Figure 5B).

Next, we added c-di-GMP to the EMSA system to explore its effect on the binding activity of HpoR to the *iniBAC* promoter. Strikingly, with increasing concentrations of c-di-GMP (2.5–400 μM), the binding activity of HpoR gradually decreased (Figure 5C, lanes 3–7), indicating that high levels of c-di-GMP alleviated the binding activity of HpoR to the *iniBAC* promoter. A chromatin immunoprecipitation (ChIP) assay was carried out to further assess the effect of a high level of c-di-GMP on the *iniBAC*p-binding activity of HpoR in the *M. bovis* BCG strain. The *ydeH*-*hishpoR* or *ydeH*(mut)*-hishpoR* co-overexpression plasmids were electroporated into the wild-type *M. bovis* BCG strain to obtain a strain with a high level of c-di-GMP (BCG/*ydeH*-*hishpoR*) and a control strain (BCG/*ydeH*(mut)-*hishpoR*). The HisHpoR protein was precipitated by a His antibody, and then the precipitated HisHpoR-bound DNA products were analyzed and quantified by qPCR. The results indicated that the *iniBAC*p-binding activity of HpoR was significantly lower in the *ydeH*-overexpressing strain than in the *ydeH*(mut)-overexpressing strain (Figure 5D), consistent with the RT-qPCR results (Figure 5A).

In general, elevated c-di-GMP levels alleviate HpoR DNA-binding ability to the *iniBAC* promoter, thereby stimulating the expression of *iniBAC* in *M. bovis* BCG.

### 3.6. c-di-GMP Positively Regulates Multidrug Resistance of M. bovis BCG

Our previous results showed that c-di-GMP reversed HpoR-mediated inhibition of the expression of *iniBAC* and that *iniBAC* negatively regulated *M. bovis* BCG multidrug susceptibility by enhancing envelope permeability. Therefore, we further investigated the drug susceptibility of the strain with elevated levels of c-di-GMP. Growth assays under drug-free conditions and under 0.04 μg/mL INH, 0.5 μg/mL EMB, 0.025 μg/mL Str, and 0.01 μg/mL RIF stress showed that the bacterial counts of the BCG/*ydeH* strain were significantly greater than those of the BCG/*ydeH*(mut) strain (Figure 5E–I).

Since c-di-GMP regulates drug susceptibility through *iniBAC*, and *iniBAC* is an INH-inducible gene cluster, we explored whether the intracellular c-di-GMP level responds to INH stress in *M. bovis* BCG. As shown in Figure S4, compared to that in the wild-type strain under no INH stress, intracellular c-di-GMP significantly accumulated in the BCG/WT strain under 10 μg/mL INH stress (Figure S4). Nevertheless, given the semi-quantitative nature of the ELISA, this finding should be considered preliminary until corroborated by MS-based absolute quantification.

In conclusion, our results indicated that elevated levels of c-di-GMP inhibit the DNA-binding activity of HpoR, alleviating its inhibitory effect on *iniBAC* expression and thereby maintaining envelope permeability and reduced multidrug susceptibility in *M. bovis* BCG.

### 3.7. The INH Susceptibility Regulatory Pathway Is Conserved in Mycobacteria

Our work revealed a new regulatory pathway for multidrug susceptibility in *M. bovis* BCG; elevated c-di-GMP levels upregulate *iniBAC* expression by reducing HpoR-mediated inhibition, thereby reducing multidrug susceptibility. To determine whether the multidrug susceptibility regulatory pathway also exists in *M. tuberculosis*, we investigated the homology between the *M. bovis* BCG, *M. tuberculosis,* and *M. smegmatis* HpoR proteins by aligning their amino acid sequences. The data showed that the *M. bovis* BCG and *M. tuberculosis* HpoR proteins were 100% identical. Meanwhile, the *M. bovis* BCG and *M. smegmatis* HpoR proteins shared 74.3% identity (Figure S5). In addition, the *iniBAC* genes and promoter were 100% identical in *M. bovis* BCG and *M. tuberculosis*. Our research also demonstrated that HpoR was a conserved c-di-GMP receptor in mycobacteria [26].

In summary, HpoR exhibits high sequence conservation in the mycobacterial genus, strongly suggesting that this regulatory model is conserved. Nevertheless, functional validation in *M. tuberculosis* and other pathogenic species will be necessary to confirm this notion.

## 4. Discussion

The mycobacterial cell wall serves as a natural barrier against drug-induced damage and is also a crucial target for antituberculosis drugs [34]. INH is converted into isonicotinic acid, which inhibits mycolic acid synthesis and generates bactericidal reactive oxygen species (ROS), collectively killing *M. tuberculosis* cells [35]. Gene expression analysis under INH pressure has revealed that mycobacteria regulate INH susceptibility through multiple mechanisms. Upregulation of *inhA* expression to promote mycolic acid synthesis can rescue cell wall damage [36], while inhibition of the tricarboxylic acid cycle and promotion of the triacylglycerol pathway reduce metabolic activity and intracellular ROS levels [37,38]. Additional mechanisms include regulation of redox homeostasis through downregulation of ndh (encoding NADH dehydrogenase) to reduce NAD production and induction of the oxidative stress enzyme AhpC to protect bacteria from oxidative damage [39,40]. INH also induces upregulation of drug efflux pump genes such as efpA to reduce intracellular drug accumulation [40]. EMB, another antituberculosis drug, inhibits the synthesis of another cell wall component, arabinogalactan [41]. Previous research revealed that EMB inhibited InhA activity, downregulates mycolic acid synthesis, and acts synergistically with INH [42]. Research on EMB-related resistance mechanisms is limited, but the *embCAB* cluster is involved in arabinogalactan synthesis and its regulator EmbR affects the EMB susceptibility of *M. tuberculosis* [43]. Moreover, the resistance mechanism of EMB may interfere with INH-mediated susceptibility [42]. The *iniBAC* cluster, induced by INH and EMB, mediates INH and EMB tolerance through the IniA pump [10]. Overall, the extensive regulatory mechanisms of drug signaling and susceptibility-related genes are still poorly understood. Our study establishes a direct link between the c-di-GMP-HpoR axis and INH tolerance, with the IniBAC cluster identified as the key downstream effector.

The *iniBAC* cluster broadly responds to cell wall damage and multidrug stress [9,15]. The IniR-regulated expression of several drug resistance-related genes, including *iniBAC*, affects multidrug resistance [17]. Previous reports have shown that IniR responds to ATP and trehalose to promote *iniBAC* expression [13,15]. However, the mechanism of transcriptional regulation and drug susceptibility of the *iniBAC* cluster is not fully understood. We found that HpoR represses *iniBAC* transcription, thereby increasing envelope permeability; conversely, disruption of this repression reduces DPH uptake, suggesting enhanced barrier function of the envelope, which likely accounts for the broad-spectrum tolerance observed. In summary, our results support that the *iniBAC* gene cluster reduced mycobacterial drug susceptibility by maintaining envelope permeability.

**Figure 6:**
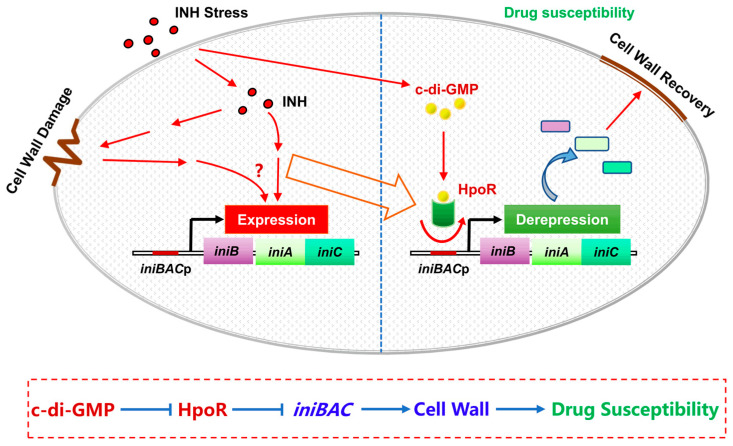
Model of c-di-GMP-mediated multidrug susceptibility and the regulatory signaling pathway in mycobacteria. Upon INH stress, elevated c-di-GMP levels alleviate HpoR-mediated repression of *iniBAC* transcription, leading to increased expression of IniBAC proteins, which in turn reduces envelope permeability and thereby decreases susceptibility to multiple antibiotics. The DPH-based permeability assay serves as the primary readout for barrier function in this model; further structural studies are warranted to assess cell-wall architecture. Blue arrows: *iniBAC* gene translation into IniBAC protein, question mark: whether INH directly regulates *iniBAC* expression is unknown, dashed line: unresolved regulatory pathway, red: increased expression intensity and green: reduced expression intensity.

In addition, we found that upon INH challenge, c-di-GMP accumulation relieves HpoR-mediated repression, establishing a feed-forward loop that reinforces envelope permeability. It should be noted that our assessment of cell-envelope properties relies primarily on the DPH-binding assay, which measures the penetration of a hydrophobic fluorescent probe into the bacterial membrane and thus serves as a reliable proxy for envelope permeability rather than a direct readout of structural cell-wall integrity (e.g., peptidoglycan thickness, arabinogalactan crosslinking, or mycolic acid composition). Furthermore, we demonstrated that c-di-GMP enabled *M. bovis* BCG to reduce drug susceptibility by reversing the inhibitory effect of HpoR on the target cluster *iniBAC*. Thus, our study was suggestive of a transcriptional regulatory pathway that regulates multidrug susceptibility and extends the regulatory function of c-di-GMP to mycobacterial drug susceptibility regulation. In this study, ELISA was used to detect intracellular c-di-GMP, which is simple and high-throughput, but has limitations: antibody specificity may be insufficient, extraction and purification steps may affect recovery rates, and ELISA only provides relative quantification. Therefore, although the data can show the increasing trend upon ydeH overexpression and INH treatment, the precise values should be interpreted with caution, and future LC-MS/MS validation for absolute quantification is required (Figure 6).

In summary, our research constructed a transcriptional regulatory model in which c-di-GMP-mediated HpoR regulates the expression of *iniBAC* to affect envelope permeability and control multidrug susceptibility. Future work will be required to test whether this pathway directly governs the bactericidal efficacy of INH and EMB in pathogenic *M. tuberculosis* using genetically engineered clinical strains. With the widespread use of antibiotics and the success of first-line anti-TB drugs, some progress has been made in treating tuberculosis. However, owing to the emergence of various multidrug-resistant strains, as a result of incomplete treatment, multidrug-resistant *M. tuberculosis* has become a cause for concern. Therefore, new drug targets must be identified. Understanding the multidrug resistance mechanism of *M. tuberculosis* and exploring the drug resistance regulatory pathway in mycobacteria represent promising directions for future research. 

We acknowledge that our experimental evidence is currently restricted to *M. bovis* BCG and *M. smegmatis*—well-established surrogate models for pathogenic mycobacteria. Although the high sequence conservation of HpoR and the *iniBAC* promoter implies that this regulatory circuitry may operate in *M. tuberculosis* [26], caution is warranted when extrapolating these results to clinical isolates or tuberculosis chemotherapy.

Nevertheless, our discovery that the c-di-GMP-HpoR axis transcriptionally gates *iniBAC* expression introduces a previously unrecognized layer of regulation that links a ubiquitous bacterial second messenger to envelope integrity. Whether this pathway contributes to the non-genetic, phenotypic tolerance observed in a subpopulation of *M. tuberculosis* during antibiotic treatment—as opposed to genetically fixed resistance—warrants further investigation using pathogenic strains and appropriate infection models. Until such validation is performed, the translational potential of this pathway (e.g., as a target for adjunctive therapy) remains speculative. Our study primarily provides a refined mechanistic framework for understanding transcriptional adaptation to cell-wall stress in the mycobacterial model system.

## Data Availability

The original contributions presented in the study are included in the article. Further inquiries can be directed to the corresponding authors.

## Supplementary Materials

The following supporting information can be downloaded at: https://www.mdpi.com/article/10.3390/microorganisms14071579/s1, Figure S1: Detection of the level of c-di-GMP in *Mycobacterium bovis* BCG strains; Figure S2: Proteomic analysis of the effect of HpoR on protein expression of *M. bovis* BCG; Figure S3: EMSA assays for the binding motif sequence of *iniBACp* recognized by HpoR; Figure S4: Detection of the level of c-di-GMP in *M. bovis* BCG strains upon INH stress; Figure S5: Conservation analysis of the HpoR among *M. bovis* BCG, *Mycobacterium tuberculosis* and *Mycobacterium smegmatis*; Table S1: The primers used in this study; Table S2: Proteomics assays for the protein differential analysis between *hpoR*-deleted and wild-type strain of *M. bovis* BCG.
