# Supplementary material for: The Cyclic Di-GMP Receptor HpoR Modulates Mycobacterial Multidrug Susceptibility by Regulating IniBAC-Mediated Envelope Permeability
Source: Microorganisms. 2026 Jul 20;14(7):1579. doi: 10.3390/microorganisms14071579 (PMC13413693; doi:10.3390/microorganisms14071579)
Supplement: Supplementary file 1 [file microorganisms-14-01579-s001.zip › microorganisms-4397325-supplementary.pdf]

The Cyclic Di-GMP Receptor HpoR Modulates Mycobacterial Multidrug Susceptibility by Regulating IniBAC-Mediated Envelope Permeability

Xiao Liu [1,2,†], Xiaocui Ling [1,2,†], Kun Wang [1], Jiachen Zheng [1], Hao Li [1], Minhao Guo [1], Yanzhe Ou [1], Jie Lu [1,*] and Weihui Li [1,*]

1 State Key Laboratory for Conservation and Utilization of Subtropical Agro-Bioresources, Guangxi Technology Innovation Center for Microbial Resources Development and Utilization, College of Life Science and Technology, Guangxi University, Nanning 530004, China

2 College of Life Science and Technology, Central South University of Forestry and Technology, Changsha 410004, China

* Correspondence: jlu@gxu.edu.cn (J.L.); lwhlbx@163.com (W.L.); Tel.: +86-771-2852965 (W.L.)

† These authors contributed equally to this work.

A list of the supplementary materials:

Figure S1. Detection of the level of c-di-GMP in *Mycobacterium bovis* BCG strains.

Figure S2. Proteomic analysis of the effect of HpoR on protein expression of *M. bovis* BCG.

Figure S3. EMSA assays for the binding motif sequence of *iniBAC*p recognized by HpoR.

Figure S4. Detection of the level of c-di-GMP in *M. bovis* BCG strains upon INH stress.

Figure S5. Conservation analysis of the HpoR among *M. bovis* BCG, *Mycobacterium tuberculosis* and *Mycobacterium smegmatis*.

Table S1 The primers used in this study.

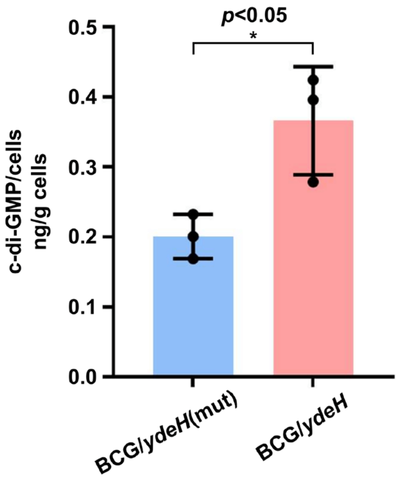

Figure S1 Detection of the level of c-di-GMP in *Mycobacterium bovis* BCG strains. Detection of the intracellular concentration of c-di-GMP in the BCG/*ydeH* and BCG/*ydeH*(mut) *M. bovis* BCG strains. Data were presented as mean ± SD for three biological replicates. The intracellular levels of c-di-GMP were converted to nanogram per gram (wet weight cells). Two-tailed Student's *t*-test was performed for statistical analysis (*p = 0.0264).

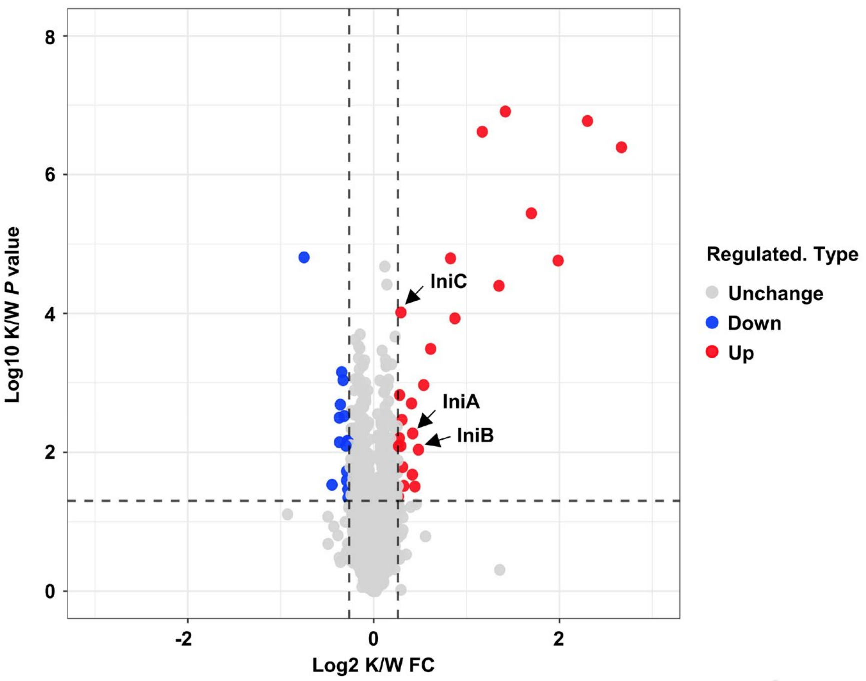

Figure S2 Proteomic analysis of the effect of HpoR on protein expression of *M. bovis* BCG. Volcanic diagram of protein expression difference between the Δ*hpoR* strain (K) and wild-type strain (W) was determined by proteomic analysis. The significantly upregulated proteins were shown by red spots. Downregulated and nonsignificant change proteins were shown by blue and grey spots, respectively.

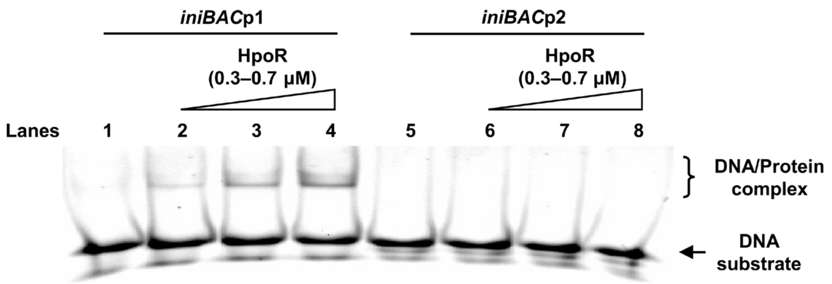

***iniBAC*p1:CGAGGACGGGGGATTGCCGACAGACCATCCGGCTGTCTGGAACCACCCGGTCGTTGACC**

***iniBAC*p2: CGAGGACGGGGGATTGCCGAATATACATCCGGCTATATAGAACCACCCGGTCGTTGACC**

Figure S3 EMSA assays for the binding motif sequence of *iniBAC*p recognized by HpoR. Wild-type short *iniBAC*p (*iniBAC*p1) DNA substrate (lanes 2-4) and mutant *iniBAC*p2 (lanes 6-8) were co-incubated with 0.3-0.7 μM HpoR, Lanes 1 and lanes 5 only *iniBAC* promoter DNA. The reaction mixture was loaded on the gel for detection and analysis.

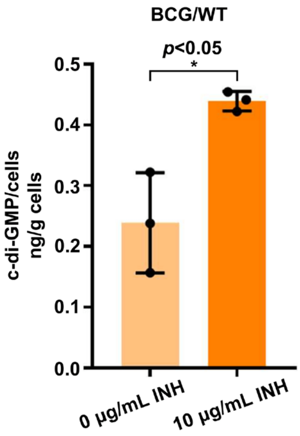

Figure S4 Detection of the level of c-di-GMP in *M. bovis* BCG strains upon INH stress. Assays for the intracellular concentration of c-di-GMP in wild-type *M. bovis* BCG (BCG/WT) strain upon INH stress. The intracellular levels of c-di-GMP were converted to nanogram per gram (wet weight cells). Two-tailed Student's *t*-test was performed for statistical analysis (*$p = 0.0146$).

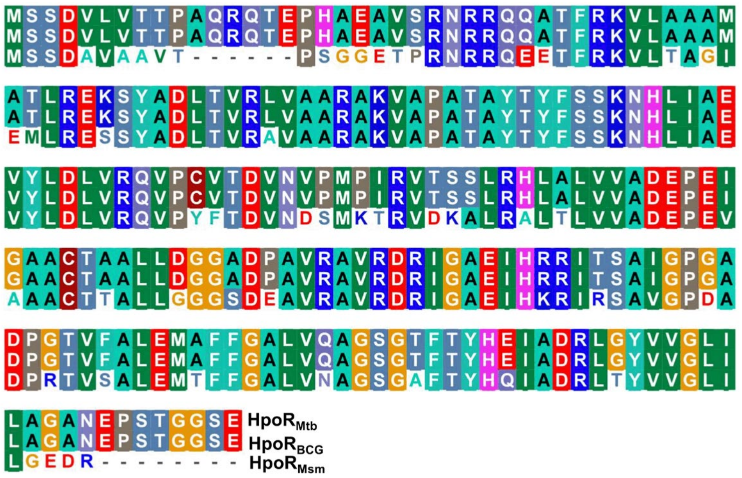

Figure S5 Conservation analysis of the HpoR among *M. bovis* BCG, *Mycobacterium tuberculosis* and *Mycobacterium smegmatis*. Amino acid sequence alignment of HpoR among *M. bovis* BCG, *M. tuberculosis* and *M. smegmatis*.

Supplementary table

Table S1. The primers used in this study

Name	Sequence 5' to 3'	General description
*Eco*R I *ydeH* f	ACGC GAATTC ATGATCAAGAAGACAACG	*ydeH*, *ydeH*(mut)
Xba I *ydeH* r	ACGC TCTAGA TTAAACTCGGTTAATCAC	overexpression
hpoR f	ATATGGATCCGCGTGTCCAGCGATGCAGTG	*hpoR* overexpression
hpoR r	AGATTCTAGATCAT CGGTCCTCTCCCAGGA	
RT*iniB* f	TCGTCGTGGGAACTGGAGGC	RT-PCR analysis for
RT*iniB* r	CGCCGTAGAACACCACCGACAT	the expression of
RT*iniA* f	CGACTACATCTACGACGAGGCG	*iniBAC*
RT*iniA* r	GCAGCACCAGTCGTCGGGAA	
RT*iniC* f	GGGAACCCGCTGGGAGGACA	
RT*iniC* r	GCCGGGACACCTCGACCATG	
RT*sigA* f	CGAGGAAGAAGAAGCTGATG	
RT*sigA* r	CGTCTTTGCGTGCCTGTC	
B iniB f	GCGCGGATCCATGACCTCGCTTATCGATTAC	*iniBAC*
X iniC r	GCCGTCTAGATCAGCGGCGAGCAGAGAACT	overexpression
*iniBAC*p	GACACCCACCGATCCGCGCCCCTTC	
*iniBAC*p	TGGCCGCCGCGTTAGGGGATTAG	EMSA assay
*bcg3074c*p	TCCACGTGGACGGCGCCGCC	
*bcg3074c*p	GACCCGGCCGTCCGGACCCG	
Kpn I *his* f	ATTCGGTACCATGGGCAGCAGCCATCATCA	
E ydeH f	ACGC*GAATTC*ATGATCAAGAAGACAACG	ChIP assay
X ydeH r	ACGC*TCTAGA*TTAAACTCGGTTAATCAC	
*E iniBAC*p f	ATTA*GAATTC*GACACCCACCGATCCGCGCCCCTTC	
*X iniBAC*p r	ATTA*TCTAGA*CTGGCCGCCGCGTTAGGGGATTAG	The β-galactosidase
*E bcg3074c*p f	TATAAT*GAATTC*TCCACGTGGACGGCGCCGCC	activity assay
*X bcg3074c*p r	TATAAT*TCTAGA*GACCCGGCCGTCCGGACCCG	

Table S2 Proteomics assays for the protein differential analysis between *hpoR* -deleted and wild-type strain of M. bovis BCG

Protein accession	Protein description	K/W Ratio	K/W P value	Gene name	Regulated Type
A0A0H3M8R7	Probable dehydrogenase/reductase OS=Mycobacterium bovis (strain BCG / Pasteur 1173P2) GN=BCG_0821	6.356	4.0407E-07	BCG_0821	Up
A0A0H3M2B3	Probable aldehyde dehydrogenase NAD dependant aldA OS=Mycobacterium bovis (strain BCG / Pasteur 1173P2) GN=aldA	4.932	1.6897E-07	aldA	Up
A0A0H3MB27	Possible 4-carboxymuconolactone decarboxylase OS=Mycobacterium bovis (strain BCG / Pasteur 1173P2) GN=BCG_0823	3.962	1.73319E-05	BCG_0823	Up
A0A0H3M4D7	Probable dehydrogenase/reductase OS=Mycobacterium bovis (strain BCG / Pasteur 1173P2) GN=BCG_0822	3.245	3.612E-06	BCG_0822	Up
A0A0H3M8A6	Fatty-acid-CoA ligase fadD26 OS=Mycobacterium bovis (strain BCG / Pasteur 1173P2) GN=fadD26	2.673	1.2318E-07	fadD26	Up
A0A0H3M8R3	Cytochrome P450 51 cyp51 OS=Mycobacterium bovis (strain BCG / Pasteur 1173P2) GN=cyp51	2.548	3.9994E-05	cyp51	Up
A0A0H3M7U0	Phenolpthiocerol synthesis type-I polyketide synthase ppsA OS=Mycobacterium bovis (strain BCG / Pasteur 1173P2) GN=ppsA	2.25	2.4176E-07	ppsA	Up
A0A0H3M301	Uncharacterized protein OS=Mycobacterium bovis (strain BCG / Pasteur 1173P2) GN=BCG_0814c	1.834	0.000117751	BCG_0814c	Up
A0A0H3MDP0	Phenolpthiocerol synthesis type-I polyketide synthase ppsB OS=Mycobacterium bovis (strain BCG / Pasteur 1173P2) GN=ppsB	1.775	1.60893E-05	ppsB	Up
A0A0H3M9X8	Probable thioesterase tesA OS=Mycobacterium bovis (strain BCG / Pasteur 1173P2) GN=tesA	1.53	0.00032411	tesA	Up
A0A0H3MA45	Heat shock protein hsp OS=Mycobacterium bovis (strain BCG / Pasteur 1173P2) GN=hsp	1.453	0.00107648	hsp	Up
A0A0H3MAC3	Isoniazid inductible gene protein iniB OS=Mycobacterium bovis (strain BCG / Pasteur 1173P2) GN=iniB	1.397	0.009179	iniB	Up
A0A0H3M829	Putative cyclopropane-fatty-acyl-phospholipid synthase ufaA1 OS=Mycobacterium bovis (strain BCG / Pasteur 1173P2) GN=ufaA1	1.361	0.031158	ufaA1	Up
A0A0H3M1Z8	Isoniazid inductible gene protein iniA OS=Mycobacterium bovis (strain BCG / Pasteur 1173P2) GN=iniA	1.338	0.0053622	iniA	Up
A0A0H3MBT6	Uncharacterized protein OS=Mycobacterium bovis (strain BCG / Pasteur 1173P2) GN=BCG_1057c	1.335	0.021001	BCG_1057c	Up
A0A0H3MII2	Carbonic anhydrase OS=Mycobacterium bovis (strain BCG / Pasteur 1173P2) GN=canB	1.327	0.00198135	canB	Up
A0A0H3M4B9	Uncharacterized protein OS=Mycobacterium bovis (strain BCG / Pasteur 1173P2) GN=BCG_1268	1.253	0.030484	BCG_1268	Up
A0A0H3MD97	Uncharacterized protein OS=Mycobacterium bovis (strain BCG / Pasteur 1173P2) GN=BCG_1624	1.241	0.0163007	BCG_1624	Up
A0A0H3M214	Uncharacterized protein OS=Mycobacterium bovis (strain BCG / Pasteur 1173P2) GN=BCG_0663A	1.235	0.003415	BCG_0663A	Up
A0A0H3M1A8	Isoniazid inductible gene protein iniC OS=Mycobacterium bovis (strain BCG / Pasteur 1173P2) GN=iniC	1.226	9.676E-05	iniC	Up
A0A0H3M771	PE family protein OS=Mycobacterium bovis (strain BCG / Pasteur 1173P2) GN=PE25	1.225	0.0081008	PE25	Up
A1KKR8	3-methyl-2-oxobutanoate hydroxymethyltransferase OS=Mycobacterium bovis (strain BCG / Pasteur 1173P2) GN=panB	1.214	0.00149981	panB	Up
A0A0H3M9U2	Uncharacterized protein OS=Mycobacterium bovis (strain BCG / Pasteur 1173P2) GN=ltp2	1.212	0.0062631	ltp2	Up
A0A0H3M5U4	Uncharacterized protein OS=Mycobacterium bovis (strain BCG / Pasteur 1173P2) GN=BCG_1909	1.204	0.0081398	BCG_1909	Up
A0A0H3M506	Putative D-amino acid oxidase aao OS=Mycobacterium bovis (strain BCG / Pasteur 1173P2) GN=aao	1.203	0.043322	aao	Up
A0A0H3MCE1	Probable lipoprotein lprE OS=Mycobacterium bovis (strain BCG / Pasteur 1173P2) GN=lprE	0.827	0.0070217	lprE	Down
A0A0H3M199	Probable transcriptional regulatory protein OS=Mycobacterium bovis (strain BCG / Pasteur 1173P2) GN=BCG_0114	0.826	0.045299	BCG_0114	Down
A0A0H3M2S1	Uncharacterized protein OS=Mycobacterium bovis (strain BCG / Pasteur 1173P2) GN=BCG_0238c	0.825	0.034242	BCG_0238c	Down
A0A0H3M4I2	Putative undecapaprenyl-phosphate alpha-n-acetylglucosaminyltransferase rfe OS=Mycobacterium bovis (strain BCG / Pasteur 1173P:	0.822	0.0068361	rfe	Down
A0A0H3M9U5	Phosphatidate cytidylyltransferase OS=Mycobacterium bovis (strain BCG / Pasteur 1173P2) GN=cdsA	0.817	0.025544	cdsA	Down
A0A0H3MA30	Probable phosphotriesterase php OS=Mycobacterium bovis (strain BCG / Pasteur 1173P2) GN=php	0.817	0.0187795	php	Down
A0A0H3M775	Ribokinase OS=Mycobacterium bovis (strain BCG / Pasteur 1173P2) GN=rbsK	0.813	0.0080434	rbsK	Down
A0A0H3M3T9	Probable gaba permease gabP (4-amino butyrate transport carrier) OS=Mycobacterium bovis (strain BCG / Pasteur 1173P2) GN=gabl	0.803	0.0030041	gabP	Down
A0A0H3M8S0	Probable conserved exported protein OS=Mycobacterium bovis (strain BCG / Pasteur 1173P2) GN=BCG_0826c	0.796	0.00091615	BCG_0826c	Down
A0A0H3MGQ7	Phenolpthiocerol synthesis type-I polyketide synthase ppsD OS=Mycobacterium bovis (strain BCG / Pasteur 1173P2) GN=ppsD	0.788	0.00070065	ppsD	Down
A0A0H3M7R7	Possible transcriptional regulatory protein (Possibly tetR/acrR-family) OS=Mycobacterium bovis (strain BCG / Pasteur 1173P2) GN=I	0.78	0.002057	BCG_0367	Down
A0A0H3M9R4	Probable conserved transmembrane transport protein mmpL10 OS=Mycobacterium bovis (strain BCG / Pasteur 1173P2) GN=mmpL1	0.775	0.0071642	mmpL10	Down
A1KET4	Phosphoheptose isomerase OS=Mycobacterium bovis (strain BCG / Pasteur 1173P2) GN=gmhA	0.774	0.0031845	gmhA	Down
A0A0H3MA28	Possible conserved protein OS=Mycobacterium bovis (strain BCG / Pasteur 1173P2) GN=BCG_0262	0.733	0.029425	BCG_0262	Down
A0A0H3M8B0	Phenolpthiocerol synthesis type-I polyketide synthase ppsE OS=Mycobacterium bovis (strain BCG / Pasteur 1173P2) GN=ppsE	0.595	1.55536E-05	ppsE	Down
